# The impact of vitamin D, vitamin C, and zinc supplements on immune status among Jordanian adults during COVID-19: cross-sectional study findings

**DOI:** 10.1186/s12889-023-17172-8

**Published:** 2023-11-16

**Authors:** Hala K. Nawaiseh, Dana N. Abdelrahim, Hayder Al-Domi, Mohammad S. AL-Assaf, Furat K. AL-Nawaiseh

**Affiliations:** 1 Department of nutrition and food technology, Faculty of Agriculture, The University of Jordan, 11942 Amman, Jordan; 2 Research Institute for Medical and Health Sciences, Sharjah University, 27272 Sharjah, United Arab Emirates; 3 Department of Ears, Nose and Throat, King Hussein Medical Centre (KHMC), Amman, Jordan; 4 Jordan Center for Disease Control (JCDC), Amman, Jordan

**Keywords:** Immunity-status-score, Supplements, Corona-Virus, Ascorbic-acid

## Abstract

**Background and aims:**

Nutritional status is essential for the maintenance of the immune system, with malnutrition suppressing immunity. The aims of the current study were to assess the immune status of a group of Jordanian adults and to evaluate the association between vitamin C, vitamin D, and zinc consumption and the Immune Status during the COVID-19 pandemic.

**Methods:**

A total of 615 adults Jordanian participants were enrolled in this study, an online- based cross sectional survey was used as a tool for this study. Data was collected by distributing the questionnaire form link through social media platforms. The association between ISQ score and the supplement intake pattern (daily, weekly, monthly and rarely) was assessed using multinomial logistic regression analysis, described as Odds ratio and 95% CI.

**Results:**

Data have indicated that the majority of the participants did not take Vitamin D supplements during the pandemic (46.3%). Also, there was a significant association between the frequency of Vitamin D supplement intake and ISQ (r = 12.777; P < 0.05). Data showed that the majority of participants used vitamin C supplementation (49.4%). Also, there was a significant association between the frequency of Vitamin C supplement intake and ISQ (r = 12.797; P < 0.05). Data also have indicated that the majority of the participants did not increase their consumption of Zinc during the COVID-19 pandemic (55.6%).

**Conclusions:**

The findings of this study suggest a significant association between the frequency of Vitamin D, and vitamin C supplement intake and ISQ. Nutritional status is essential for the maintenance of the immune system, with malnutrition suppressing immunity.

## Introduction

COVID-19 remains a worldwide problem [1]. With nearly 170,000 reported fatalities to date, the coronavirus disease 2019 (COVID-19) pandemic has affected nearly 2.5 million people worldwide [2]. Malnutrition, particularly deficiencies in vitamins A, D, C, zinc, and selenium, has been linked to COVID-19 infection [1]. Numerous studies have shown that during the COVID-19 epidemic, people in the Middle East consumed more dietary supplements and herbal remedies to ward off the disease. [3]. To protect patients, it is also important to use evidence-based practices to manage nutritional supplements an herbal items [3]. Supplementation with vitamins C and D, as well as with zinc and selenium, for people who are at risk of respiratory viral infections or for those who are nutrient deficient, has been emphasized as potentially useful [4].

Elderly people, African Americans, obese people, and institutionalized people are disproportionately affected by coronavirus disease 2019 (COVID-19), which is caused by severe acute respiratory syndrome coronavirus 2 (SARS-CoV-2) [5]. These groups have also been identified as high-risk populations for vitamin D deficiency [5]. In addition to vitamin D playing a crucial role in the metabolism of calcium and phosphate, it is known for its biological effects on immunological regulation [6].

Vitamin C has been shown to improve immunity, wound healing, energy metabolism, and nervous system function [7]. A healthy and effective host defence mechanism requires vitamin C, and it is thought that administering vitamin C pharmacologically can improve immune performance [2]. Previous research has demonstrated that vitamin C inhibits the replication of some viruses, including influenza, poliovirus, and herpes simplex virus [2]. Vitamin C may be useful in treating viral infections and possibly COVID-19 [2].

Zinc, the second most prevalent trace metal in the body after iron, is crucial for many cellular processes, including the preservation of immune function [8]. Zinc is an anti-inflammatory and antioxidant micronutrient [8]. In some clinical trials on COVID-19, it was proven that zinc has a well-established role in immunity, and it is currently being used [8]. The scant research on the benefits of zinc supplementation do not support its effectiveness [9]. Therefore, COVID-19 prevention or treatment strategies based on selenium or zinc supplementation are currently unjustified [9].

Nutritional status is essential for the maintenance of the immune system, with malnutrition suppressing immunity [10]. Specific essential nutrients are correlated with viral infection and mortality from COVID-19 [11]. Vitamin D, vitamin C, and the trace element zinc are known to support immune function [11]. Data have indicated that deficiencies and suboptimal nutritional status of these micronutrients can potentially decrease resistance to infections and reinfections. [12]. Therefore, considering the effects of the COVID-19 pandemic and the controversial evidence, this research aimed to assess the immune status of a group of Jordanian adults and to evaluate the association between vitamin C, vitamin D, and zinc consumption and the Immune Status during the COVID-19 pandemic.

## Methodology

### Study protocol

To assess the nutritional status, immune status, and dietary intake of specific types of nutrients during the COVID-19 pandemic, a total of 615 adult Jordanian participants were enrolled in this study, and an online-based cross-sectional survey was conducted. All healthy Jordanian adults aged 18–65 years old were eligible to participate in this study, except cancer patients, who were excluded. Data were collected from June 2021 to November 2021 by distributing the questionnaire form link through social media platforms.

### Data collection tools

The online questionnaire instrument contained the following domains: (1) sociodemographic data, (2) dietary intake of specific nutrients, (3) diagnosis of COVID-19, and (4) immune status. The participants’ sociodemographic data included age, sex, education, type of job, height and weight and history of any disease.

### Dietary intake questionnaire

The dietary intake section asked semiqualitative dietary intake questions for 15 food items (the sources of vitamin D, C and zinc in a normal healthy diet), the frequency of vitamin C, D and zinc supplementation during the COVID-19 pandemic and the dose of consumption. The diagnosis of COVID-19 section asked whether the participants or their families had previously or currently had COVID-19 and whether they were vaccinated or planned to be vaccinated.

The choices for the vitamin D supplementation questions were recategorized as follows: from no intake to less than 1 per month was considered “rarely”, 1–3 times per month was considered “monthly”, 1 time per month, 2–4 times per week and 5–6 times per week were considered “weekly”, and once daily up to 6 times or more daily were considered “daily”. The choices for the zinc supplementation questions were recategorized as follows: no intake was considered “rarely”, 1–2 times per month was considered “monthly”, 1 time per month and 2–3 times per week were considered “weekly”, and once daily up to 2–3 times daily was considered “daily”. The choices for the vitamin C supplementation questions were recategorized as follows: rarely was defined as “monthly”, sometimes was defined as “weekly”, and approximately every day was defined as “daily”.

The actual content of one serving of each food item that included vitamin D, zinc and vitamin C was obtained from ESHA SQL food analyser software. Total consumption was calculated by multiplying the actual content of one serving of food by the frequency of consumption and recategorized. For the questions that contained more than one food item, the average content of all items was used as the actual content of each supplement.

#### The Immune Status Questionnaire (ISQ)

The Immune Status Questionnaire (ISQ) is a valid and reliable 7-item scale used to assess perceived immune status over the past 12 months. The ISQ asks about “sudden high fever”, “diarrhoea”, “headache”, “skin problems (e.g., acne and eczema)”, “muscle and joint pain”, “common cold” and “coughing”. The responses were collected through 5-point Likert scale descriptors (“never”, “sometimes”, “regularly”, “often” and “(almost) always)”. Then, a total score was obtained, and a cut-off value was applied (< 6) to determine the status of immune functioning and differentiate between those with poor and normal immune health [13].

Each item of the Immune Status Questionnaire (ISQ) was scored as follows: never = 0 points; sometimes = 1 point; regularly = 2 points; often = 3 points; and (almost) always = 4 points; then, the sum score of the 7 ISQ items was calculated. The raw score was recoded to obtain the final ISQ score (where 0 = very poor and 10 = excellent perceived immune status), and the ISQ final score was recoded by the cut-off score for reduced immune functioning, which was ISQ < 6, and a value greater than or equal to 6 was considered good immune functioning [13]. The Arabic version of the questionnaire was used in the data collection process [13, 14].

### Data identification and statistical analysis

Participants’ response data were encoded and analysed using IBM SPSS Statistics, version 26.0. Sociodemographic continuous data are presented as the means and standard deviations. The categorical variables are expressed as frequencies and percentages of observed values. The questions on vitamin D, zinc and vitamin C intake are described using frequencies and percentages. The correlation between supplement intake and the ISQ score was assessed using correlation analysis. Subsample analysis was performed for the questions on the diagnosis and history of COVID-19 using the independent sample t test and 95% confidence intervals (CIs). Additionally, an independent sample t test was performed to assess the relationship between the ISQ score and disease occurrence.

The association between the ISQ score and the supplement intake pattern (daily, weekly, monthly and rarely) was assessed using multinomial logistic regression analysis, described as odds ratios and 95% CIs. Total supplement consumption was compared to the dietary recommendations by age and sex groups using a t test. The sex-specific effect was assessed in comparison with the ISQ score using crosstabs, chi-square tests and risk assessment analysis. Body mass index (BMI) was calculated by the weight and height of participants and categorized using the WHO definition. Then, the correlation between BMI categories and ISQ score was evaluated for both sexes. All the data significance levels were set at *P* < 0.05.

## Results

### Sociodemographic characteristics

A total of 615 participants from different areas in Jordan were recruited in this study; 80.2% were females. A greater proportion were aged 18–29 (47.6%) and had a bachelor’s degree (67.0%). A greater proportion of participants had jobs in education (39.2%), followed by medical jobs (36.7%). The majority of the participants had a normal body weight (48.0%). The majority of participants with chronic disease were obese (34.3%), and the smallest proportion of the participants were diagnosed with cancer (0.5%) (Tables 1 and 2).

**Table 1:** Sociodemographic and health characteristics of the study participants (n = 615)

Variable	N (%)	Male N (%)	Female N (%)
Gender	(n = 615)		
Male	122 (19.8%)	-	-
female	493 (80.2%)	-	-
Age groups	(n = 615)	(n = 122)	(n = 493)
18–29 year.	293 (47.6%)	57 (46.7%)	236 (47.9%)
30–39 year.	156 (25.4%)	17 (13.9%)	139 (28.2%)
40–49 year.	109 (17.7%)	26 (21.3%)	83 (16.8%)
50–59 year.	46 (7.5%)	20 (16.4%)	26 (5.3%)
≥ 60 year.	11 (1.8%)	2 (1.6%)	9 (1.8%)
Education	(n = 613)	(n = 120)	(n = 493)
Secondary	29 (4.7%)	8 (6.6%)	21 (4.3%)
Diploma	49 (8.0%)	5 (4.1%)	44 (8.9%)
B.Sc.	412 (67.0%)	74 (60.7%)	338 (68.6%)
Higher education	123 (9.7%)	33 (27.0%)	90 (18.3%)
Work	(n = 540)	(n = 110)	(n = 430)
Educational Jobs	241 (39.2%)	46 (37.7%)	195 (39.6%)
Handicrafts Jobs	73 (11.9%)	30 (24.6%)	43 (8.7%)
Medical Jobs	226 (36.7%)	34 (27.9%)	192 (38.9%)
BMI (kg/m^2^)	(n = 604)	(n = 122)	(n = 482)
Underweight	27 (4.4%)	1 (0.8%)	26 (5.3%)
Normal	295 (48.0%)	46 (37.7%)	249 (50.5%)
Overweight	176 (28.6%)	46 (37.7%)	130 (26.4%)
Obese class I	74 (12.0%)	19 (15.6%)	55 (11.2%)
Obese class II	25 (4.1%)	9 (7.4%)	16 (3.2%)
Obese class III	7 (1.1%)	1 (0.8%)	6 (1.2%)
Diseases Occurrence	Percent of Occurrence from the Total (n = 615)		
Diabetes	27 (4.4%)		
Hypertension	45 (7.3%)		
Atherosclerosis	5 (0.8%)		
Cancer	3 (0.5%)		
Obesity	211 (34.3%)		

^The categorical variables were described using frequencies and percentages of observed values^

**Table 2:** Anthropometric Measurements of the study participants (n = 615)

Variable	Mean ± S.D.	Minimum	Maximum
**Weight (Kg)**	69.74 ± 16.12	40.0	170.0
**Height (cm)**	1.65 ± 0.08	1.43	1.99
**BMI (kg/m** ^**2**^ **)**	25.51 ± 5.11	16.23	53.65

^The continuous data were presented as mean and standard deviation^

Table 3 shows the vitamin D supplementation status and its correlation with ISQ score. The majority of the participants did not take vitamin D supplements during the pandemic (46.3%). The highest proportion of the participants took vitamin D supplements once during the last month (39.7%), and the majority used a 50,000-mg dose (30.1%). The majority of the participants indicated that they did not increase their consumption of vitamin D during the COVID-19 pandemic (57.2%). The data indicated a significant association between the frequency of vitamin D supplement intake and ISQ score (r = 12.777; P < 0.05).

**Table 3:** Vitamin D supplement status & correlation with ISQ score

Variable	Categories	N (%)	Correlation (P-value)
**Do you take vitamin D supplement?**	Yes, continuously	81 (13.2%)	
	Yes	285 (46.3%)	
	No	249 (40.5%)	
**How many times do you take vitamin D supplement in last month?**	No intake	119 (19.3%)	12.777 (0.026) *
	1 time	244 (39.7%)	
	2 times	57 (9.3%)	
	3 times	31 (5.0%)	
	4 times	124 (20.2%)	
	More than that	40 (6.5%)	
**What is the dose of vitamin D that you use?**	No intake	124 (20.2%)	2.534 (0.771)
	1000 mg	174 (28.3%)	
	2000 mg	41 (6.7%)	
	5000 mg	76 (12.4%)	
	10,000 mg	15 (2.4%)	
	50,000 mg	185 (30.1%)	
**Did your consumption of vitamin D increase during COVID-19?**	Yes	263 (42.8%)	
	No	352 (57.2%)	

^The categorical variables were described using frequencies and percentages of observed values. The correlation between the supplement intake and the ISQ score was assess using correlation analysis. P−value < 0.05 considered significant^

Table 4 shows the zinc and vitamin C supplement status of the participants. The data showed that the majority of participants used vitamin C supplementation (49.4%). Moreover, the participants’ consumption of vitamin C supplements increased during the COVID-19 pandemic (57.7%). Most of the participants took vitamin C supplements once during the last month (73.2%), and the greatest proportion of participants used a 50-mg dose (30.1%). The data indicated a significant association between the frequency of vitamin C supplement intake and ISQ score (r = 12.797; P < 0.05). However, there was no significant association between the dose of vitamin C and the ISQ score (P > 0.05). The data indicated that the majority of the participants did not increase their consumption of zinc during the COVID-19 pandemic (55.6%).

**Table 4:** Zinc & vitamin C supplements status & correlation with ISQ score

Variable	Categories	N (%)	Correlation (P-value)
**Did your consumption of Zinc increase during COVID-19?**	Yes	273 (44.4%)	
	No	342 (55.6%)	
**Do you take vitamin C supplement?**	Yes, continuously	49 (8.0%)	
	Yes	304 (49.4%)	
	No	262 (42.6%)	
**How many times do you take vitamin C supplement in last month?**	No intake	136 (22.1%)	12.797 (0.046) *
	1 time	450 (73.2%)	
	2 times	16 (2.6%)	
	3 times	4 (0.7%)	
	4 times	5 (0.8%)	
	5 times	3 (0.5%)	
	6 times	1 (0.2%)	
**What is the dose of vitamin C that you use?**	No intake	132 (21.5%)	5.775 (0.329)
	50 mg	185 (30.1%)	
	100 mg	85 (13.8%)	
	250 mg	30 (4.9%)	
	500 mg	98 (15.9%)	
	1,000 mg	85 (13.8%)	
**Did your consumption of vitamin C increase during COVID-19?**	Yes	355 (57.7%)	
	No	260 (42.3%)	

^The categorical variables were described using frequencies and percentages of observed values. The correlation between the supplement intake and the ISQ score was assess using correlation analysis. P−value < 0.05 considered significant^

Table 5 shows the association between the frequency of food item consumption and the ISQ score in the study population. A significant effect was reported for only one food item, nuts, and consuming nuts on a daily basis may have significantly (P < 0.05) led to good immune functioning by 63%. The intake of no other food item had a significant effect on immune functioning (P > 0.05). Table 6 shows the average intake of each of the micronutrients for the whole population. Compared to the ISQ score, this table indicates that there was no significant association (P > 0.05) between vitamin D, zinc or vitamin C and the ISQ score.

**Table 5:** Association between the frequency of food items consumption and ISQ score among the study participants

Food item	Serving	Daily	weekly	monthly	rarely	P-value
Vitamin D sources						
**Cornflakes supp. with Vitamin D**	0.75 cup					0.460
Good immune functioning		13	92	54	220	
Reduced immune functioning		13	45	29	148	
OR (95% CI)		1.632 (0.657–4.052)	2.187 (0.793–6.026)	3.181 (1.195–8.471)	1	
**Orange juice supp. with Vitamin D**	1 cup					0.155
Good immune functioning		45	139	56	139	
Reduced immune functioning		23	79	34	99	
OR (95% CI)		0.852 (0.455–1.595)	0.759 (0.366–1.573)	0.970 (0.515–1.828)	1	
**Cooked liver**	0.5 cup					0.633
Good immune functioning		1	39	76	263	
Reduced immune functioning		2	20	45	168	
OR (95% CI)		3.318 (0.291–37.82)	3.254 (0.279–37.95)	3.658 (0.301–44.40)	1	
**Sardine fish**	0.5 cup					0.274
Good immune functioning		6	36	50	287	
Reduced immune functioning		3	15	33	184	
OR (95% CI)		0.320 (0.035–2.88)	0.317 (0.034–2.981)	0.416 (0.042–4.09)	1	
**Egg**	1 egg					0.955
Good immune functioning		49	239	49	42	
Reduced immune functioning		34	143	29	29	
OR		1.179 (0.562–2.47)	1.159 (0.566–2.374)	1.082 (0.620–1.89)	1	
**Butter supp. with Vitamin D**	1 tsp					0.125
Good immune functioning		7	55	39	278	
Reduced immune functioning		11	38	22	164	
OR (95% CI)		2.965 (0.946–7.68)	3.454 (1.065–11.20)	2.414 (0.789–7.387)	1	
Zinc sources						
**Brown rice, brown bread & Burghul**	1 serving					0.594
Good immune functioning		236	83	35	25	
Reduced immune functioning		151	52	16	16	
OR (95% CI)		0.976 (0.472–2.017)	1.478 (0.731–2.991)	1.231 (0.779–1.946)	1	
**Legumes**	0.5 cup					0.781
Good immune functioning		49	234	82	14	
Reduced immune functioning		37	139	47	12	
OR (95% CI)		0.627 (0.240–1.636)	1.175 (0.633–2.181)	1.182 (0.702–1.99)	1	
**Nuts**	1/3 cup					0.009*
Good immune functioning		102	184	75	18	
Reduced immune functioning		43	117	60	15	
OR (95% CI)		0.631 (0.273–1.462)	0.604 (0.343–1.065)	0.692 (0.432–1.109)	1	
**Peanut butter & sesame butter (Tahina)**	2 Tbsp					0.410
Good immune functioning		33	87	93	166	
Reduced immune functioning		14	55	59	107	
OR (95% CI)		0.563 (0.268–1.183)	0.577 (0.261–1.277)	0.560 (0.256–1.223)	1	
**Boiled or fried egg**	1 egg					0.141
Good immune functioning		91	217	43	28	
Reduced immune functioning		43	139	35	18	
OR (95% CI)		0.969 (0.424–2.217)	0.557 (0.294–1.056)	0.627 (0.393-1.00)	1	
**Meat**	100 g					0.487
Good immune functioning		41	188	35	115	
Reduced immune functioning		27	118	28	62	
OR (95% CI)		1.086 (0.563–2.092)	0.662 (0.296–1.482)	0.861 (0.467–1.589)	1	
Vitamin C sources						
**Citrus fruits**	2 serving				**-**	0.571
Good immune functioning		178	176	25	**-**	
Reduced immune functioning		108	107	20	**-**	
OR (95% CI)		0.793 (0.393–1.604)	0.997 (0.680–1.461)	1	**-**	
**Tomato, sweet pepper**	2 serving				**-**	0.286
Good immune functioning		230	137	12	**-**	
Reduced immune functioning		140	78	17	**-**	
OR (95% CI)		0.414 (0.174–0.983)	1.139 (0.767–1.691)	1	**-**	
**Cruciferous vegetables**	2 serving				**-**	0.348
Good immune functioning		37	254	88	**-**	
Reduced immune functioning		22	149	64	**-**	
OR (95% CI)		0.580 (0.284–1.185)	0.686 (0.355–1.322)	1	**-**	

^AOR was adjusted for age, gender and BMI.cat. OR, odds ratio; CI, confidence interval. P−value < 0.05 considered significant^

**Table 6:** Correlation of total nutrient daily intake (from all food items) (after ESHA entries) with ISQ score

Nutrients	Intake	Correlation with ISQ score	P-value
Vitamin D	1.99 mcg	0.061	0.132
Zinc	3.23 mg	0.008	0.843
Vitamin C	185.81 mg	-0.030	0.459

^The correlation between the average intake and the ISQ score was assess using correlation analysis. P−value < 0.05 considered significant^

## Discussion

Nutritional status is essential for the maintenance of the immune system, with malnutrition suppressing immunity [10]. It has been reported that malnutrition is associated with an increased risk of SARS-CoV-2 infection, severity, and mortality [10]. Nutritional status and specific essential nutrients are correlated with viral infection and mortality from COVID-19 [11]. Vitamin D, vitamin C, and the trace element zinc are known to support immune function [11]. In the current study, different patterns of dietary supplement use were identified in a group of individuals during the COVID-19 pandemic in Jordan.

The majority of the participants did not take vitamin D supplements during the pandemic (46.3%). Moreover, the greatest proportion of the participants took vitamin D supplements once during the last month (39.7%), and the majority used a dose of 50,000 mg (30.1%). However, the majority of the participants reported that they did not increase their consumption of vitamin D during the COVID-19 pandemic (57.2%). Our findings also indicated a significant association between the frequency of vitamin D supplement intake and ISQ score (r = 12.777; P < 0.05). In parallel to the current findings, data from an online cross-sectional questionnaire among Japanese adults indicated that most participants (91.7%) reported not currently using dietary supplements for the prevention of SARS-CoV-2 infection; however, only 8.3% reported that they used it as a therapeutic tool [1]. The current data are similar to the data from studies conducted through online surveys in Lebanon, the Kingdom of Saudi Arabia, Palestine, Jordan, and the United Arab Emirates, which indicated that only 21.3% of respondents agreed that nutritional supplements may minimize the risk of being infected with COVID-19; however, 45.4% believed that dietary supplements have therapeutic effects against COVID-19, and only 15.2% recognized that dietary supplements are useful only in cases of deficiencies [15]. The most common supplements used were vitamin C (77.8%), vitamin D (55.7%), and zinc (42.9%). [15]However, there were increases in the use of antioxidants (14% vs. 15.6%), vitamin C (35.3% vs. 42.1%), vitamin D (35.5% vs. 41%), vitamin E (15.2% vs. 17.5%), and zinc (18.8% vs. 29.3%) [16]. However, data from a cross-sectional web-based survey in the United Arab Emirates (UAE) population indicated that 56.6% of participants reported using dietary supplements to prevent or cure COVID-19, with vitamin C (84.5%), vitamin D (31.6%), and multivitamins (17%) being the most commonly reported supplements [17]. In a randomized control trial, the administration of two weeks of oral supplementation of vitamin D (1000 UI vs. 5000 UI) to patients with suboptimal vitamin D status was associated with fast recovery from cough and sensory loss among those who received a greater amount [19]. On the basis of these results, it seems appropriate to prescribe vitamin D as an adjuvant to COVID-19 treatment for individuals with mild to severe symptoms [19].

Vitamin D is efficient at alleviating SARS-CoV-2 infection, according to a retrospective observational analysis of SARS-CoV-2 positivity in relation to serum 25(OH) D concentration measurements in the United States [18]. Data indicated that SARS-CoV-2 positivity in those with a serum concentration of 55 ng/mL was roughly half that of those with a serum value of 20 ng/ml. Data from a meta-analysis of 43 observational studies with a total of 612,601 patients indicated that among subjects with vitamin D deficiency (a serum 25(OH)D concentration < 20 ng/ml), the risk for COVID-19 infection was higher compared to those with serum 25(OH)D concentrations > 30 ng/mL (OR, 1.26; *P* < 0.01) [6]. Additionally, data from a large cohort study conducted in the United Kingdom indicated that supplementation with omega-3 fatty acids, probiotics, multivitamins, or vitamin D was related to a reduced risk of infection with the COVID-19 virus; however, vitamin C, zinc, or garlic supplementation did not show therapeutic effects against COVID-19 [19].

The therapeutic effects of vitamin D in relation to COVID-19 are due to its potential immunomodulatory effects, such as maintenance of epithelial cell integrity, promotion of antimicrobial peptides, modulation of antigenic presentation by dendritic cells, promotion of anti-inflammatory cytokines, and regulation of renin production [20]. The other function of vitamin D is to reduce the possibility of a cytokine storm, which is linked to COVID-19-induced acute respiratory distress syndrome and may cause considerable multiorgan damage [21]. The nutritional status of people regarding vitamin D may have been exacerbated during the COVID-19 pandemic because of limitations in mobility, which may suppress normal immune functions [11, 22]. Recent research has demonstrated that vitamin D regulates and suppresses the cytokine inflammatory response that causes acute respiratory distress syndrome observed in severe and frequently fatal COVID-19 infections [23]. It has been recommended to maintain serum 25(OH)D concentrations between 40 and 60 ng/ml [24]. However, to achieve these concentrations, an adult would require 4000 to 6000 IU/day of vitamin D [24]. Elderly people and those who already have chronic diseases stand to gain the greatest benefits [24].

To maximize the immunological response, it has even been suggested that micronutrient RDAs can be exceeded through supplementation as long as care is taken not to exceed the upper intake limits [4].

Our results indicated that the majority of participants used vitamin C supplementation (49.4%). Moreover, the participants’ consumption of vitamin C supplements increased during the COVID-19 pandemic (57.7%). The majority of participants took vitamin C supplements once during the last month (73.2%), and the greatest proportion of the participants used a dose of 50 mg (30.1%). The data indicated a significant association between the frequency of vitamin C supplement intake and ISQ score (r = 12.797; P < 0.05). However, there was no significant association between the dose of vitamin C and the ISQ score (P > 0.05). In parallel to the current findings, a cross-sectional study in Saudi Arabia reported that approximately 22% of the 5258 participants reported that they had used herbal and nutritional supplements to prevent the disease during the epidemic [25]. In addition, vitamin C was the most often utilized supplement to boost immunity and reduce the likelihood of developing COVID-19 [25]. Similarly, a cross-sectional study that included 1460 participants aged between 12 and 86 years in Riyadh, Saudi Arabia, demonstrated a considerable increase in the intake and frequency of nutritional supplements during the COVID-19 pandemic period compared to the period preceding the COVID-19 pandemic (P = 0.000). Moreover, the majority of participants reported utilizing vitamin C (56%) [3]. Due to its essential function in innate (nonspecific) and acquired (specific) immunity, vitamin C is one of the most widely utilized vitamins across different communities [26, 27]. Additionally, a study among Lithuanian adults reported that the majority of respondents (73.7%) said that the pandemic had no impact on their usage of dietary supplements, and one-fourth of respondents’ consumption increased (24.6%) [28].

Data from a randomized controlled trial among patients with severe COVID-19 infection in Tehran, Iran, found that an intervention with 6 g/day of vitamin C for 5 days among 30 patients with severe COVID-19 did not affect COVID-19-related symptoms, including body temperature, peripheral capillary oxygen saturations (SpO2), length of ICU admission, or mortality, compared to a placebo control group. [7].

Previous research that included 17 patients with confirmed COVID-19 found that three days of vitamin C supplementation (1 g/8 h) was associated with decreased inflammatory markers in all hospitalized patients [2]. Moreover, in a study among 46 patients, a greater dose (6 g every 12 h on Day 1 and 6 g for the next four days) decreased the risk of mortality and improved oxygen support [29]. A cross-sectional, questionnaire-based study among adult patients (≥ 18 years) in Saudi Arabia found that some dietary supplements were consumed by participants even before their infection with COVID-19, which may indicate their belief in the protective and immune-boosting effects of these supplements, including vitamin C (48.8%) [30].

Although vitamin C deficiency was observed in COVID-19 patients (110) and could be utilized to reduce susceptibility to alleviate respiratory tract infections, there are inadequate data to support its efficacy in protecting people from SARS-CoV-2 infection [31, 32]. Human infections, degenerative diseases, oral diseases, and behavioural disorders are all more common when zinc is deficient [33]. Current information indicates a link between lower zinc intake and COVID-19 disease severity [33, 34].

This study found that more than half (55.6%) of the study sample did not increase their intake of zinc supplements during COVID-19. A study performed among elderly individuals, who are the most vulnerable age group to be infected with COVID-19 and documented in the NHANES III, agreed with our study results and indicated that approximately 35-45% of elderly people consumed much less zinc than recommended [33]. A study performed among 935 Polish residents reported that consumption of zinc and vitamin D was increased among participants with higher education levels (59%) compared with noneducated or participants with lower education levels and increased among participants with a medical background compared with participants with backgrounds in other fields (54.5%) [35]. Another study in Egypt demonstrated that only 5.6% of the participants consumed zinc supplements during the pandemic [36].

This study found that the average daily intake of zinc was 3.23 mg, which had a very weak correlation with the ISQ score (0.008) and was not significant (P > 0.05). This correlation is still largely debated in many studies. A study conducted in Japan among 62 patients with COVID-19 showed a strong association between low serum zinc levels and the severity of the disease, which can be justified by the fact that zinc may reduce viral replication and increase the immune response by providing an additional shield against the initiation and progression of COVID-19 [37, 38]. Another study performed in the United States found a correlation between zinc supplementation and the mitigation of COVID-19 severity when they gave 10-, 25-, and 50-mg daily doses, which may explain why in our results, no correlation was shown since the intake was much less than one-third of the minimum given dose in the study [39]. Furthermore, a meta-analysis performed in Iran suggested that zinc supplementation is associated with a lower risk of mortality among COVID-19 patients [40]. In addition, a Bangladeshi study confirmed that the duration of vitamin D, C and zinc supplementation and medication was significantly associated with reduced patient hospitalization [41].

## Conclusion

In this study, 46.3% of the participants did not take vitamin D supplements during the pandemic. Additionally, the current data indicated a significant association between the frequency of vitamin D supplement intake and ISQ scores. Regarding zinc consumption, 55.6% of the participants did not consume zinc supplements, and the average daily intake of zinc was 3.23 mg, which had a very weak correlation with the ISQ score. These nutrients might be beneficial for health by helping to avoid excess nutritional intake, even though there is no scientific evidence that they can help prevent SARS-CoV-2 infection. Data have indicated that deficiencies and suboptimal nutritional status of these micronutrients can potentially decrease resistance to infections and reinfections. This study has some strengths and limitations that should be acknowledged. To the best of our knowledge, this is the first study in Jordan to assess the use of vitamin D, vitamin C, and zinc supplements and its association with ISQ scores in a group of Jordanian individuals during the COVID-19 pandemic. In addition, one of the characteristics of online surveys is their efficacy in reaching people who live in different geographical areas and, moreover, the speed of data collection. However, there are a few limitations that need to be considered while interpreting the results of the present study. First, the major limitation of the current study is the self-report nature of the data obtained. Therefore, response biases associated with over-reporting and under-reporting could be included in the dataset. Secondly, we recruited our subjects via social networks using a non-probability snowball sampling technique. This created selection bias, which may have limited the generalizability of the current results. Thirdly, Due to the cross-sectional nature of the study design, exact causality between the studied variables cannot be determined. Finally, the small sample size affects the representativeness of the sample to the population of Jordan.

## Data Availability

The data that support the findings of this study are available upon request from the corresponding author. The data cannot be publicly available due to privacy and ethical considerations.
